# High-School Teachers’ Beliefs about Effort and Their Attitudes toward Struggling and Smart Students in a Confucian Society

**DOI:** 10.3389/fpsyg.2016.01366

**Published:** 2016-09-14

**Authors:** Shun-Wen Chen, Bih-Jen Fwu, Chih-Fen Wei, Hsiou-Huai Wang

**Affiliations:** ^1^Institute of Learning Sciences, National Tsing Hua UniversityHsinchu, Taiwan; ^2^Center for Teacher Education, National Taiwan UniversityTaipei, Taiwan; ^3^Department of Psychology and Counseling, University of TaipeiTaipei, Taiwan

**Keywords:** beliefs about effort, Confucian culture, implicit theory of intelligence, role obligation

## Abstract

Previous studies conducted in Western societies showed that instructors’ beliefs about intellectual ability affected their attitudes toward students. However, in many East Asian societies influenced by Confucian culture, teachers not only hold beliefs of ability but also two kinds of beliefs about effort: *obligation-oriented belief* (i.e., believing that effort-making is a student’s role obligation) and *improvement-oriented belief* (i.e., believing that effort can conquer the limitations of one’s ability). This study aimed to investigate the relationships between teachers’ effort beliefs and their attitudes toward favoritism, praise, and expectations toward struggling and smart students. The participants were 151 Taiwanese high-school teachers. Results of Structure Equation Modeling showed that (1) teachers’ obligation-oriented belief about effort was positively correlated with their favoritism, praise, short-term and long-term expectations of struggling students, but negatively correlated with their favoritism and praise of smart students, (2) teachers’ improvement-orientated belief about effort was negatively correlated with their short-term expectation of smart students and favoritism of struggling students, but positively correlated with their praise of smart students, and (3) the entity theory of intelligence was negatively correlated with favoritism and praise of struggling students, but positively correlated with favoritism of smart students. The theoretical and cultural implications are discussed.

## Introduction

Just as scientists develop theories to interpret the phenomena they investigate, laypersons may develop theories or beliefs about ability and effort. For example, some people believe that a person’s ability is something that he/she cannot change much. Others may believe that anyone can improve his/her ability by exerting effort. Furthermore, some people may hold the belief that even if the ability cannot be changed much, one still has a duty to work hard when pursuing certain goals. In the present paper, we argue that, especially in a society influenced by the Confucian cultural heritage (e.g., Taiwan), people tend to emphasize the value of effort in pursuing specific goals with high social expectations, such as pursuing academic achievements. When pursuing those goals, people may hold two beliefs about effort: an *obligation-oriented belief* (i.e., believing that effort-making is one’s role obligation) and an *improvement-oriented belief* (i.e., believing that effort can conquer the limitations of one’s ability). We investigated the relationship between teachers’ effort beliefs and their attitudes about favoritism, praise, and expectations toward struggling and smart students.

### The Implicit Theory of Intelligence

Previous studies showed that people’s beliefs about effort and ability may influence their learning motivation. Dweck and Leggett (1988), Dweck et al. (1995), Dweck (1999), and Hong et al. (1999) proposed a model to explain the relationships between learning motivation and the implicit belief in intellectual ability. According to this model, people may hold different implicit theories about the nature of intelligence. Some believe that intelligence is more of an unchangeable, fixed entity (i.e., an entity theory). Others think of intelligence as a malleable quality that can be developed (i.e., an incremental theory). Many studies conducted in Western societies have shown that students’ implicit theories of intelligence may affect their learning motivation. In sum, those holding an entity theory, unlike those holding an incremental theory, tend to draw conclusions about their academic ability from setbacks and are more likely to give up or withdraw effort when faced with difficulty (Dweck and Leggett, 1988; Dweck, 1999; Hong et al., 1999; Heine et al., 2001; Blackwell et al., 2007).

Hong et al. (1999) argued that different implicit theories of intelligence are associated with distinct frameworks or “meaning systems.” Therefore, a belief in intellectual ability can affect not only learners’ motivation but also teachers’ attitudes toward their students. In a simulation experiment (Rattan et al., 2012), undergraduate participants first read an article that manipulated their implicit theories of math intelligence. Then they took the role of a seventh grade math teacher and were asked about their attitudes toward a simulated student who scored 65% on the first test of the year. Results showed that instructors holding an entity (versus incremental) theory were more likely to both comfort the student for his/her low math ability (e.g., explain that not everyone is meant to pursue a career in this field) and use “kind” strategies which were unlikely to promote engagement with math (e.g., assigning less homework). In a follow-up study, graduate students who were actually math-related instructors or teaching assistants in undergraduate courses were recruited. The participants were told to imagine that they were working as teaching assistants for an introductory course in their department and asked about their attitudes toward a simulated student who had received a failing grade on the first test of the course. The results were the same; instructors who held a more entity (versus incremental) theory readily expressed significantly lower expectations of this students’ future performance based on one low test score and endorsed the comforting and potentially unhelpful practices (e.g., talking to the student about dropping the class).

### Two Beliefs about Effort in East Asian Societies

Most studies of the dichotomous model of entity versus incremental theory and its consequences have been conducted in Western societies (Dweck and Leggett, 1988). Whether this model can be generalized to East Asian societies (e.g., Taiwan, Hong Kong, Korea, and Japan) is an issue required further investigated. Under the influences of Confucian cultural traditions, parents, and teachers in those East Asian societies generally place a tremendous emphasis on the importance of their children or students’ academic achievements. Many high school students in those countries attend cram schools to improve their performance of exams, which influence or even determine they can get into top universities (Crystal and Stevenson, 1991; Morris and Sweeting, 1995; Li, 2012). It has been reported that the primary obligations of children and adolescents in Hong Kong, Taiwan, and Korea are considered to be to study hard and to excel in academic performance (Hong, 2001; Li, 2012). Apparently, East Asians place strong emphasis on making effort to achieve academic goals. We argue that, in order to understand the psychology and behaviors of East Asian people pursuing such goals, it is necessary to use the emic approach of cultural psychology to analyze the internal meanings and values within a cultural system (Hong et al., 1999; Hwang, 2012).

Many East Asian societies are culturally and historically rooted in the Confucian tradition, which has a meaning system stressing role-obligations, effort-making, and academic achievement (Chen et al., 2009; Li, 2012; Fwu et al., 2014). The thoughts and behaviors of a virtuous person, as depicted in the Confucian doctrines, should be in accordance with his/her social roles (Ames, 2011; Hwang, 2012), such as the beliefs that “a son should obey his parents” or “a student should study hard.” Chen et al. (2009) proposed a “framework of Chinese achievement goals” and argued that, in many East Asian societies, people are expected to continuously expend great effort to achieve a special kind of goals: *vertical goals*. Vertical goals are achievements with high social expectations and are related to the obligation of one’s social roles. The performances of individuals in their pursuit of these goals are ranked into a vertical ladder of achievement by others. Individuals are usually obliged to meet the expectations of significant others, such as parents, and compete with their peers to climb up the “achievement pyramid” (Fwu et al., 2014). In many East Asian societies influenced by Confucian values, pursuing academic achievement is often regarded as a student’s vertical goal (King and McInerney, 2014).

Some cultural psychologists (Chang, 2000; Shweder, 2000; Hwang, 2012) argued that the operationalization of the psychological constructs had better to be contextualized via the cultural meaning system. However, the influential meaning system of values in a society usually coexists with social institutions (Archer, 1995). For over 1000 years (from ~600 AD to 1905), China implemented an “imperial examination system” (kē-jǔ) to select government officials. On the one hand, this system assessed scholars on their knowledge of traditional Confucian classics and instilled Confucian values into the mind of the general public for generations. On the other hand, the system in turns was regarded as an effective method by Confucian scholars to select and promote talented and virtuous persons to be officials (Chan, 2014). Consequently, this examination system not only established the influence of Confucianism, but also became a dominant and fair way for ordinary people to acquire high social status (Hwang, 2012; Li, 2012). The impacts of high-stakes exam and Confucian cultural system may vary among East Asian societies (Park, 2010; Brown and Wang, 2015), however, academic achievement which is usually assessed by exams is still an important vertical goal in Taiwan nowadays (Chen et al., 2009).

Because pursuing vertical goals is regarded as an obligation of one’s social role, people tend to believe that it is one’s duty to exert oneself and that effort-making is the most important way to improve their performance in the pursuit of such goals. Li (2012) argued that, in the Confucian tradition, the meaning of “learning” entails role obligation and improvement of oneself. A “good” student is a one who has a positive image, one who has the qualities of diligence, earnestness, sincerity, perseverance, steadfastness, and endurance of hardship in learning. These characteristics are all synonymous with “effort” and could be termed as “learning virtues.” In other words, “to study hard” is regarded as the obligation of a student. Previous study found that the duty conceptions were strong predictors for Asian students on academic performance (Peterson et al., 2013).

Moreover, effort-making is regarded as a necessary means to improve one’s learning. As many popular Chinese proverbs describe, “Learning is like rowing upstream; not to advance is to drop back (xúe rú nì shuǐ xíng zhoū, bù jìn zé tuì),” “practice makes perfect (shú néng shēng qiǎo),” “effort can make up for inability (qín néng bǔ zhuō),” “With persistence, an iron pestle can be ground down to a needle (tiě chǔ mó chéng zhēn).” These beliefs are the reasons why many East Asian parents and teachers constantly encourage their children or students to make effort in academic learning, even if the pupils are already performing well (Li, 2012).

Therefore, we argue that, in societies influenced by the Confucian tradition, people will develop two important beliefs about effort: *obligation-oriented* and *improvement-oriented* beliefs. In academic learning, to hold the obligation-oriented belief about effort is to believe that it is a student’s role obligation to make effort in learning. To hold the improvement-oriented belief about effort is to believe that effort can conquer the limitations of one’s ability and improve one’s academic performance. Furthermore, under the influence of cultural values and from the experiences of their daily lives, laypersons may develop not only one dimension but multiple beliefs about effort and ability at the same time. Hong (2001) found that many Chinese teachers viewed making effort as an indication of lack of intelligence, similar to the view of entity theorists on intelligence. But these teachers also believe that effort, more than intelligence, determines the outcomes of academic performance. In other words, it is possible that a teacher can believe the entity theory of intellectual ability while at the same time accepting to a certain degree the improvement-oriented belief about effort.

Teachers’ beliefs about effort and ability could influence their affective, cognitive, and behavioral attitudes toward learners. Few studies have investigated the relationships between teachers’ beliefs about intellectual ability and their attitudes toward students (Hong, 2001; Rattan et al., 2012), and no studies to date have aimed to investigate teachers’ obligation-oriented and improvement-oriented beliefs about effort and their relationships with the teachers’ attitudes toward students in East Asian societies. In the present study, we measured Taiwanese high-school teachers’ beliefs about effort and implicit theories of intelligence and then adopted the situation simulation method used in previous studies (Peng et al., 1997; Hong, 2001; Rattan et al., 2012) to ask participants about their attitudes toward a “struggling student” (i.e., a student who studied hard but performed mediocrely) and a “smart student” (i.e., a student who did not study hard but performed outstandingly). In addition to cognitive (i.e., short-term and long-term expectations toward students) and behavioral attitudes (i.e., praise of students), participants were also asked about their affective attitudes (i.e., tendency of favoritism) toward those students.

### Hypotheses of the Present Study

According to the framework of Chinese achievement goals (Chen et al., 2009) and the meaning system of learning virtues in Confucian culture (Li, 2012), the more a teacher believes in the obligation-oriented belief about effort, the more he/she may tend to think of “struggling students” (students who studied hard but performed mediocrely) as fulfilling a student’s role obligation and manifesting learning virtues. Moreover, teachers’ improvement-oriented belief about effort may be positively correlated with their expectations of struggling students and negatively correlated with those of “smart students” (students who did not study hard but performed outstandingly). Therefore, we hypothesized that: (1) Teachers’ obligation-oriented belief about effort was positively correlated with their affective and behavioral attitudes (i.e., favoritism and praise) toward the struggling student (H-1). (2) The improvement-orientation belief about effort was positively correlated with their short-term and long-term expectations of the struggling student (H-2). (3) The improvement-orientation belief about effort was *negatively* correlated with their short-term and long-term expectations of the smart student (H-3).

Furthermore, according to the model of the implicit theory of intelligence (Dweck and Leggett, 1988; Dweck, 1999), we hypothesized that: (4) Teachers’ entity theory of intelligence was positively correlated with their favoritism and praise of the smart student (H-4). (5) The entity theory of intelligence was *negatively* correlated with their short-term and long-term expectations of the struggling student (H-5). (6) The entity theory of intelligence was positively correlated with their short-term and long-term expectations of the smart student (H-6).

## Materials and Methods

### Participants

A total of 174 high-school teachers participated in this study. However, based on the responses of the manipulation check items, the data of 22 participants were deleted and not analyzed further. Another participant was deleted because of gender unidentified. Therefore, 151 valid samples were included in the present study (118 females; 33 males; age mean = 38.18, *SD* = 8.47).

### Procedures

All participants were asked to read and answer a questionnaire after they gave informed consents. The questionnaire was composed of two parts. The first part depicted two different students. One was a “struggling student” who studied hard but had mediocre performance. The other was a “smart student” who did not study hard but performed outstandingly. These two students were depicted by their behaviors and performances in academic achievement in the questionnaire. The terms “struggling” and “smart” did not appear in the descriptions of the two students in order to avoid conventional labeling. The description of the “struggling student” was “*Student A is not only attentive and takes notes in class but also does homework seriously and studies very hard. However, the academic performance of Student A is at roughly 35th percentile in the class.*” The description of the “smart student” was “*Student B is not attentive in class, puts little effort into homework, and does not study hard. With just a little bit of studying before exams, Student B is among the top three in the class.*”

After reading the two descriptions, participants were asked to answer four questions about their attitudes toward these two students, respectively: “*I like to be an instructor of this student*” (Favoritism), “*I will praise this student in public*” (Praise), “*I think this student will perform well on the university entrance exam*” (Short-term expectation), and “*I think this student will be an accomplished person in society*” (Long-term expectation). All items were scored on a 6-point Likert-type scale ranging from 1 (strongly disagree) to 6 (strongly agree). Participants were then asked to answer the second part of the questionnaire, which contained three scales on beliefs about effort and intelligence.

### Measures

Three items were modified from the Students’ Role-obligation Scale (Chen and Wei, 2013) to measure participants’ obligation-oriented belief about effort: “*To study hard is a student’s duty*,” “*It is a student’s responsibility to study hard*,” and “*A student should feel shame when he/she does not study hard.*” All items were scored on a 6-point Likert-type scale ranging from 1 (strongly disagree) to 6 (strongly agree).

Five items were developed to measure participants’ improvement-oriented belief about effort, e.g., “*One can improve his/her ability with no limitations,”* “*If one makes persistent efforts, his/her ability is unlimited*,” and “*Effort can conquer the limitations of one’s ability.*” All items were scored on a 6-point Likert-type scale ranging from 1 (strongly disagree) to 6 (strongly agree).

Three items were adopted from the Implicit Theory of Intelligence Scale (Dweck, 1999; Hong et al., 1999; Molden and Dweck, 2006) to measure participants’ entity theory of intelligence: “*One has a certain amount of intelligence and really cannot do much to change it*,” “*One’s intelligence is something about him/her that one cannot change very much*,” and “*One can learn new things, but one cannot really change his/her basic intelligence.*” All items were scored on a 6-point Likert-type scale ranging from 1 (strongly disagree) to 6 (strongly agree).

There were two manipulation check items in the questionnaire: “*I think Student A is smart*” and “*I think Student B is smart.*” These items were also scored on a 6-point Likert-type scale. If a participant’s response on the first item (Student A is smart) was larger than that on the second (Student B is smart), then his/her data were deleted and not analyzed further. This step was taken because such responses might not be based on the descriptions on the questionnaire or the images of the students they perceived did not match what we delivered. Thus, data on 22 participants were deleted and not analyzed in this study.

Furthermore, there were two simple (yes/no) questions in the questionnaire: “*Have you ever taught students like Student A (or Student B)?*” 98.7% of all participants gave the positive response to Student A and 82.6% to Student B. These results indicated that the descriptions of the two kinds of students might be in accordance with teaching experiences of most participants.

## Results

### Confirmatory Factor Analysis

In order to verify the reliability and validity of the scales of beliefs about effort and intelligence, a confirmatory factor analysis (CFA) of three factors model was conducted. The expectation-maximization analysis was used to estimate missing data. Results of CFA showed that the fitness of the three factors model was acceptable, χ^2^ (41) = 99.264, *p* < 0.001, χ^2^/df = 2.42, CFI = 0.913, TLI = 0.883, RMSEA = 0.097, SRMR = 0.069, gamma hat = 0.93 (Browne and Cudeck, 1993; Hu and Bentler, 1999; Fan and Sivo, 2007; Hooper et al., 2008). The values of composite reliability (CR) of three factors (Obligation-oriented belief = 0.74, Improvement-oriented belief = 0.87. Entity theory of intelligence = 0.78) were all above 0.7 (Hair et al., 1998). The values of average variance extracted (AVE) of three factors (Obligation-oriented belief = 0.49, Improvement-oriented belief = 0.58. Entity theory of intelligence = 0.56) were mostly above 0.5 (Fornell and Larcker, 1981). In sum, the CR and convergent validity of the scales were acceptable.

### Descriptive Statistics

**Table 1** shows the descriptive statistics of participants’ three beliefs about effort and intelligence. The correlation between Obligation-oriented and Improvement-oriented beliefs about effort was positively significant (*r* = 0.49, *p* < 0.001). In addition, the correlation between Obligation-oriented belief about effort and Entity theory of intelligence was also positively significant (*r* = 0.36, *p* < 0.001).

**Table 1:** Means, standard deviations, and correlation coefficients among the beliefs about effort and intelligence factors.

	Mean	*SD*	Obligation-oriented belief about effort	Improvement-oriented belief about effort
Obligation-oriented belief about effort	4.53	0.87	–	
Improvement-oriented belief about effort	4.23	1.01	0.49^∗∗∗^	–
Entity theory of intelligence	3.15	1.07	0.36^∗∗∗^	-0.03

*N = 151. ^∗∗∗^p < 0.001 (two-tailed)*.

**Table 2** shows the means and standard deviations of participants’ attitudes (i.e., Favoritism, Praise, Short-term expectation, and Long-term expectation) toward Student A (the struggling student) and Student B (the smart student). Results of 2 (students) × 4 (attitudes) within-subjects MANOVA indicated that the interaction effect was significant, *F*(3,450) = 70.10, *p* < 0.01, η^2^ = 0.318. Results of simple main effect analyses showed that participants would rather teach Student A, be more likely to praise Student A in public, and had higher expectations that Student A would be an accomplished person in society than student B (*p*s < 0.001).

**Table 2:** Means and standard deviations of participants’ Favoritism, Praise, Short-term, and Long-term expectations toward Students A and B.

	Student A		Student B	
	*M*	*SD*	*M*	*SD*
Favoritism	5.22	0.97	3.80	1.40
Praise	5.39	0.76	3.50	1.44
Short-term expectation	4.19	1.02	4.42	1.03
Long-term expectation	4.55	1.05	4.04	1.07

*N = 151.*

### Structure Equation Modeling

In order to investigate the relationships among the participants’ beliefs about effort and intelligence, an analysis of Structure Equation Modeling (SEM) was conducted. The criterion variables were the items of participants’ Favoritism, Praise, Short-term, and Long-term expectations of Student A (the struggling student) and Student B (the smart student). The predictor variables were participants’ Obligation-oriented and Improvement-oriented beliefs about effort as well as their Entity theory of intelligence. In addition, the participants’ genders and ages were included in the model as covariates.

According to the results of CFA, both Obligation-oriented and Improvement-oriented beliefs about effort were correlated with Entity theory of intelligence, therefore these two correlations were included in the SEM model (Cole et al., 2007). Furthermore, because the short-term and long-term expectations for a person should be correlated, and the correlation between participants’ Short-term and Long-term expectations for Student A was significant (*r* = 0.42, *p* < 0.001). The same result was obtained for Student B (*r* = 0.34, *p* < 0.001). Thus, these two correlations were included in the model.

**FIGURE 1:**
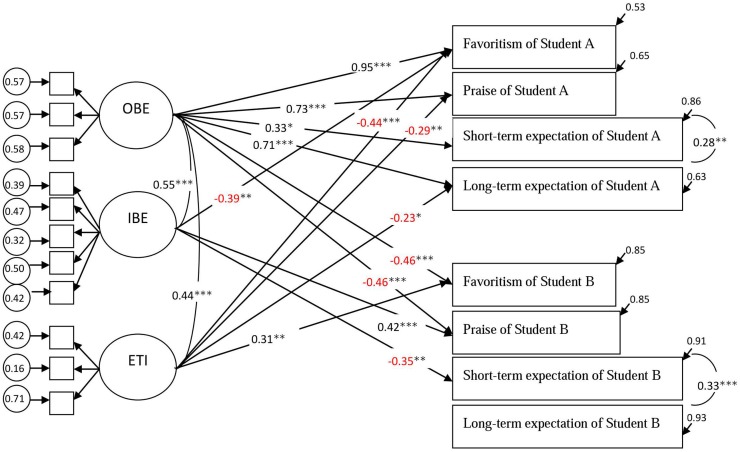
**The model of effects of participants’ beliefs about effort and intelligence on attitudes toward Student A and Student B.** OBE, obligation-oriented belief of effort; IBE, improvement-oriented belief of effort; ETI, entity theory of intelligence. Only significant paths are included in the figure. Covariates (gender and age) were not included in the figure. Standardized coefficients are reported. Negative effects were colored in red. ^∗^*p* < 0.05. ^∗∗^*p* < 0.01. ^∗∗∗^*p* < 0.001. χ^2^ (154) = 302.31, *p* < 0.001, χ^2^/df = 1.96, CFI = 0.843, TLI = 0.785, RMSEA = 0.08, SRMR = 0.075, gamma hat = 0.91.

**Figure 1** and **Table 3** shows the results of SEM analyses. The results indicated that: (1) Participants’ Obligation-oriented belief about effort was positively correlated with their Favoritism and Praise (βs = 0.95, 0.73, *p*s < 0.001, respectively) of Student A. These results supported H-1 of the present study. (2) Participants’ Improvement-orientation belief about effort was not significantly correlated with their Short-term (β = 0.07, *ns*.) and Long-term expectations (β = -0.13, *ns*.) of Student A. The H-2 was not supported. (3) Participants’ Improvement-orientation belief about effort was negatively correlated with their Short-term expectation (β = -0.35, *p* < 0.001) but not their Long-term expectation (β = -0.10, *ns.*) of Student B. These results partially supported H-3. (4) Participants’ Entity theory of intelligence was positively correlated with their Favoritism (β = 0.30, *p* < 0.01) but not Praise (β = 0.15, *ns.*) of Student B. The results partially supported H-4. (5) Participants’ Entity theory of intelligence was not significantly correlated with their Short-term and Long-term expectations of Students A (βs = -0.11, -0.23, *ns*., respectively) and Students B (βs = -0.03, 0.03, *ns*., respectively). Therefore, H-5 and H-6 were not supported.

**Table 3:** Parameter estimates and significant levels for the model.

	*b*	*SE*	*t*	*p*	β
OBE→Favoritism of Student A	1.25	0.222	5.61	^∗∗∗^	0.95
OBE→Praise of Student A	0.74	0.15	4.91	^∗∗∗^	0.73
OBE→Short-term expectation of Student A	0.45	0.19	2.43	0.015	0.33
OBE→Long-term expectation of Student A	1.01	0.21	4.91	^∗∗∗^	0.71
OBE→Favoritism of Student B	-0.86	0.27	-3.25	^∗∗∗^	-0.46
OBE→Praise of Student B	-0.90	0.28	-3.28	^∗∗∗^	-0.46
OBE→Short-term expectation of Student B	0.33	0.19	1.74	0.082	0.23
OBE→Long-term expectation of Student B	-0.02	0.19	-0.09	0.931	-0.01
IBE→Favoritism of Student A	-0.44	0.14	-3.12	0.002	-0.39
IBE→Praise of Student A	-0.13	0.10	-1.32	0.186	-0.15
IBE→Short-term expectation of Student A	0.07	0.13	0.56	0.578	0.06
IBE→Long-term expectation of Student A	-0.16	0.14	-1.17	0.243	-0.13
IBE→Favoritism of Student B	0.20	0.19	1.08	0.278	0.13
IBE→Praise of Student B	0.69	0.20	3.44	^∗∗∗^	0.42
IBE→Short-term expectation of Student B	-0.42	0.14	-2.98	0.003	-0.35
IBE→Long-term expectation of Student B	-0.12	0.14	-0.88	0.382	-0.10
ETI→Favoritism of Student A	-0.61	0.17	-3.48	^∗∗∗^	-0.44
ETI→Praise of Student A	-0.31	0.12	-2.61	0.009	-0.29
ETI→Short-term expectation of Student A	-0.16	0.15	-1.06	0.288	-0.11
ETI→Long-term expectation of Student A	-0.35	0.16	-2.21	0.027	-0.23
ETI→Favoritism of Student B	0.61	0.23	2.68	0.007	0.31
ETI→Praise of Student B	0.31	0.22	1.37	0.170	0.15
ETI→Short-term expectation of Student B	-0.05	0.16	-0.30	0.767	-0.03
ETI→Long-term expectation of Student B	0.05	0.16	0.30	0.766	0.03
Gender→Favoritism of Student A	0.017	0.169	0.103	0.918	0.007
Gender→Praise of Student A	0.093	0.134	0.697	0.486	0.051
Gender→Short-term expectation of Student A	-0.204	0.192	-1.064	0.288	-0.083
Gender→Long-term expectation of Student A	-0.274	0.182	-1.503	0.133	-0.108
Gender→Favoritism of Student B	-0.212	0.266	-0.795	0.427	-0.063
Gender→Praise of Student B	-0.365	0.273	-1.335	0.182	-0.106
Gender→Short-term expectation of Student B	-0.161	0.200	-0.808	0.419	-0.065
Gender→Long-term expectation of Student B	-0.468	0.207	-2.264	0.024	-0.181
Age→Favoritism of Student A	-0.007	0.008	-0.817	0.414	-0.059
Age→Praise of Student A	-0.011	0.007	-1.713	0.087	-0.125
Age→Short-term expectation of Student A	-0.020	0.009	-2.151	0.032	-0.167
Age→Long-term expectation of Student A	-0.027	0.009	-2.976	0.003	-0.108
Age→Favoritism of Student B	0.018	0.013	1.343	0.179	0.106
Age→Praise of Student B	0.005	0.013	0.410	0.681	0.032
Age→Short-term expectation of Student B	0.001	0.010	0.085	0.932	0.007
Age→Long-term expectation of Student B	0.015	0.010	1.521	0.128	0.122

*OBE, obligation-oriented belief of effort; IBE, improvement-oriented belief of effort; ETI, entity theory of intelligence. Gender and age were included as covariates. ^∗∗∗^p < 0.001.*

## Discussion

### Theoretical and Cultural Implications

The present study developed measurements of two beliefs about effort: obligation-oriented and improvement-oriented beliefs. Furthermore, we found that Taiwanese high school teachers’ beliefs about effort and intellectual ability had predictive effects on their attitudes toward struggling and smart students. Existing theories about learning motivations and achievement goals developed in Western cultures do not emphasize the construals of obligation-oriented and improvement-oriented beliefs about effort in academic learning. However, these two beliefs may be prevailing in many East Asian societies and have psychological and behavioral consequences.

First, the results of the present study showed that Taiwanese teachers’ obligation-oriented about effort could predict their affective, cognitive, and behavioral attitudes toward students. The obligation-oriented belief could positively predict teachers’ favoritism, praise, short-term, and long-term expectations of struggling students and negatively predict teachers’ favoritism and praise of the students who did not study hard but performed well. Note that the obligation-oriented belief of effort was a strong predictor for most of teachers’ attitudes toward the students who studied hard but performed mediocrely. The patterns of results support the framework of Chinese achievement goals (Chen et al., 2009) and the meaning system of learning virtues in Confucian culture (Li, 2012).

Second, the improvement-oriented belief about effort could positively predict teachers’ praise of the smart students, but negatively predict their short-term expectation of the same students. It’s interesting that the improvement-oriented belief could also negatively predict teachers’ favoritism toward the student who studied hard but performed mediocrely (β = -0.39, *p* < 0.01). This result may be because that the performance of the struggling students was not in line with the belief that effort can improve one’s ability. Therefore, in order to reduce the feeling of dissonance (Festinger, 1957), the more a teacher held the improvement-oriented belief about effort, the less she or he would like to teach the struggling students.

Third, our results showed that teachers’ entity theory of intelligence could negatively predict their favoritism, praise and long-term expectation toward struggling students and positively predict their favoritism toward smart students. These results are consistent with previous studies conducted in Western societies (Rattan et al., 2012). However, the results also showed that participants’ improvement-oriented belief about effort was not correlated with their entity theory of intelligence (*r* = -0.03, *ns.*). This finding may indicate that the improvement-oriented belief about effort and the entity theory of intelligence are independent construals. A previous study showed that some Chinese teachers believe that effort can facilitate the application of ability while also believing that people who have a high level of ability will not need much effort to succeed (Hong, 2001). Therefore, it is possible that some people can simultaneously believe the entity theory of intellectual ability and also accept the improvement-oriented belief about effort to a certain degree, even though these two beliefs seem contradictory on the surface. The results of the present study provided corroboration for previous researches which revealed that teachers’ beliefs can be simultaneous and contradictory (Green, 1971; diSessa, 1988; Brown, 2008).

Fourth, the results showed that the correlation between the participants’ obligation-oriented belief about effort and the entity theory of intelligence was positively significant (*r* = 0.36, *p <* 0.001). It is interesting that the predictive effects of these two beliefs on favoritism toward struggling students were both significant, but with different signs (βs = 0.95, -0.44, *p*s < 0.001, respectively). The patterns of the predictive effects were the same on praise of struggling students (βs = 0.73, -0.29, *p*s < 0.001, 0.01, respectively) and favoritism toward smart students (βs = -0.46, 0.31, *p*s < 0.001, 0.01, respectively). Similarly, the correlation between the participants’ obligation-oriented and improvement-orientated beliefs about effort was positively significant (*r* = 0.49, *p* < 0.001). The predictive effects of these two beliefs on favoritism toward struggling students were both significant, but with different signs (βs = 0.95, -0.39, *p*s < 0.001, 0.01, respectively). The patterns of their predictive effects were reverse on praise of smart students (βs = -0.46, 0.42, *p*s < 0.001, respectively). These results indicated that teachers may hold these three beliefs simultaneously and the effects of their affective and behavioral attitudes toward struggling and smart students may be opposite. In fact, we informally interviewed some teachers and asked them about their impressions and evaluations of these two kinds of students. Many teachers seemed to have mixed affects and gave uncertain responses, especially toward the students who did not study hard but performed well. On the one hand, they favored the intelligence of these students. On the other hand, they disapproved the laziness of the same students because it showed a lack of learning virtues. These phenomena may indicate that people can hold multiple beliefs about effort and ability, even though these beliefs have opposite effects on their attitudes.

### Limitations and Future Directions

The present study had several limitations. First, the items measuring each attitude on the questionnaire were few in number. Future studies could measure responses more broadly. Second, the scale of obligation-oriented belief about effort in the present study was specific to the student role and academic learning because we aimed to investigate teachers’ beliefs about the learning virtues and role obligations of students. It may be possible to develop scales in the future to measure obligation-oriented beliefs about effort in general or in other specific social contexts; e.g., at home or in the workplace. Third, we only investigated the relationships between teachers’ beliefs and their attitudes toward two kinds of students: struggling students (i.e., students who studied hard but performed mediocrely) and smart students (i.e., students who did not study hard but performed outstandingly). It would be more comprehensive if future studies could investigate other scenarios, such as students with different levels of effort and performance or students who are improving, staying the same or getting worse on effort or performance. Fourth, there were 22 participants responded in manipulation check items that the struggling student is smarter than the one who did not work hard but performed well. It is possible that these participants think “the student who works hard is smart.” However, we are not sure if these participants might misunderstand the descriptions of the two students. It would be worthwhile to explore whether individuals’ beliefs about effort and ability can influence their interpretations of “smartness” (Hong, 2001). Finally, future studies could cross-culturally compare students’ obligation-oriented and improvement-oriented beliefs about effort as well as their relationships with affects, cognitions, and behaviors.

## Author Contributions

All authors substantially contribute to the conception, analysis, and interpretation of data for the work; and revise it critically for important intellectual content; and finally approve the version to be published; and agree to be accountable for all aspects of the work in ensuring that questions related to the accuracy or integrity of any part of the work are appropriately investigated and resolved.

## Conflict of Interest Statement

The authors declare that the research was conducted in the absence of any commercial or financial relationships that could be construed as a potential conflict of interest.
